# Daytime administration of melatonin has better protective effects on bone loss in ovariectomized rats

**DOI:** 10.1186/s13018-023-03695-8

**Published:** 2023-03-23

**Authors:** Tian-lin Li, He-dong Liu, Mao-xian Ren, Zhi Zhou, Wen-kai Jiang, Min Yang

**Affiliations:** Department of Trauma Orthopedics, The First Affiliated Hospital of Wannan Medical College, Yijishan Hospital, No. 2, Zhe Shan Xi Road, Wuhu, 241001 Anhui People’s Republic of China

**Keywords:** Osteoporosis, Melatonin, Bone loss, Bone mass, Estrogen deficiency

## Abstract

**Objective:**

To explore the difference in the protective effects of intraperitoneal injection of exogenous melatonin of daytime or nighttime on bone loss in ovariectomized (OVX) rats.

**Methods:**

After bilateral ovariectomy and sham surgery, 40 rats were randomly divided into four groups: sham operation group (Sham), ovariectomy (OVX), and daytime melatonin injection group (OVX + DMLT, 9:00, 30 mg/kg/d) and nighttime injection of melatonin (OVX + NMLT, 22:00, 30 mg/kg/d). After 12 weeks of treatment, the rats were sacrificed. The distal femur, blood and femoral marrow cavity contents were saved. The rest of the samples were tested by Micro-CT, histology, biomechanics and molecular biology. Blood was used for bone metabolism marker measurements. CCK-8, ROS, and Cell apoptosis are performed using MC3E3-T1 cells.

**Results:**

Compared with treatment at night, the bone mass of the OVX rats was significantly increased after the daytime administration. All microscopic parameters of trabecular bone increased, only Tb.Sp decreased. Histologically, the bone microarchitecture of the OVX + DMLT was also more dense than the bone microarchitecture of the OVX + LMLT. In the biomechanical experiment, the femur samples of the day treatment group were able to withstand greater loads and deformation. In molecular biology experiments, bone formation-related molecules increased, while bone resorption-related molecules decreased. After treatment with melatonin administration at night, the expression of MT-1β was significantly decreased. In cell experiments, the MC3E3-T1 cells treated with low-dose MLT had higher cell viability and greater efficiency in inhibiting ROS production than the MC3E3-T1 cells treated with high-dose MLT, which in turn more effectively inhibited apoptosis.

**Conclusion:**

Daytime administration of melatonin acquires better protective effects on bone loss than night in OVX rats.

## Introduction

Osteoporosis is one of the most common bone diseases, characterized by loss of trabecular bone, destruction of bone microarchitecture, and decreased bone strength [1–3]. An estimated 10 million people have osteoporosis in the USA alone [4]. Although osteoporosis itself has no symptoms, its complications endanger the health and safety of patients [5]. Among these, fragility hip fractures are extremely destructive to the patients’ body, maybe leading to death [6–8]. Due to the high prevalence of morbidity and mortality rates of its complications, osteoporosis has caused a huge and unavoidable financial burden on the medical system [9, 10]. However, the current clinical treatment adopts lifestyle modification and the administration of bone metabolism-regulating drugs to prevent and treat osteoporosis [11]. Changing lifestyle habits requires extensive and in-depth health education and a high degree of patient compliance. Currently, bisphosphonates are widely used in the clinical treatment of osteoporosis, but the side effects, such as abdominal pain, kidney disease, and cardiovascular disease, cannot be ignored [12–15]. In addition, romosozumab, teriparatide, denosumab, and SERM are also used to treat osteoporosis. These drugs also have some reports of adverse reactions [14, 16–18]. Thus, finding an effective and safe anti-osteoporosis drug is an urgent requisite.

Melatonin (MLT, 5-hydroxy-tryptamine) is an indoleamine secreted by the pineal gland and involved in various physiological processes, such as tumor suppression, reproductive regulation, and anti-inflammatory and antioxidant reactions [19–22]. As a kind of endocrine hormone, 90% of MLT can be metabolized by the liver and excreted in the urine [23]. Several of these studies have reported that MLT is strongly associated with bone and muscle growth [24, 25]. Several studies have shown that MLT inhibits the expression of Ostrix protein by regulating the protein kinase A (PKA) and protein kinase C (PKC) signaling pathways [26]. Animal experiments revealed that MLT inhibits nuclear factor-κB receptor activator ligand (RANKL) to suppress the formation of osteoclasts but promotes Wnt/β-catenin signaling to promote the formation of osteoblasts [27]. MLT has also been under the intensive focus of clinicians because of its robust ability to regulate bone metabolism. However, because the secretion of MLT has a strong circadian rhythm, it is manifested as more secretion at night and less or no secretion during the day [28], indicating that the concentration of MLT in human blood varies greatly at different time points. This phenomenon raises an intriguing and practical question of whether injections of MLT at different time periods have varied anti-osteoporotic effects.

Presently, the efficacy of MLT for bone loss has been determined. However, due to the specificity of MLT secretion rhythm, limited studies have investigated the optimal administration time of exogenous MLT. Thus, the present study used an ovariectomized rat model to simulate the pathological characteristics of postmenopausal women. In this study, ovariectomized rats were treated with MLT for 12 weeks after bone loss. Also, various indicators in ovariectomized rats were detected using micro-computed tomography (Micro-CT), histology, biomechanics, biomarker, and molecular biology methods to evaluate the anti-osteoporosis efficacy of exogenous MLT injection at different time points during the day and night.

## Materials and methods

### Animal experiments

In this study, 40 female Sprague–Dawley (SD) rats (3-months-old, 250–300 g) were placed in an animal laboratory at 24 °C, 50% humidity, and Turn on the lights at 8:00 Beijing time and turn off at 20:00 Beijing time. This study is an original research conducted at the Yijishan Hospital Research Center of Wannan Medical College, and animal experiments were carried out at the animal experiment center of the Yijishan Hospital Research Center of Wannan Medical College. All rats were housed in SPF-level animal cages with two rats per cage. All rats were provided with unlimited access to food and water. All feed was purchased from the Qinglongshan Breeding Farm in Nanjing. All experiments were carried out in accordance with international animal welfare standards and in line with the requirements of the Animal Research Committee of Wannan Medical College (KJ2017A266).

### Animal surgery and feeding

All rats underwent animal surgery after two weeks of adaptive feeding. All rat operations were performed in a sterile operating room. All rats were randomly grouped after 2 weeks of adaptive feeding. The rats were anesthetized with pentobarbital(40 mg/kg) [29] and randomly divided into two groups: Sham surgery (Sham, n = 10) and pre-ovariectomy (OVX-ed, n = 30). The rats were placed in the prone position, the back hair was removed, and the skin was disinfected. The skin and subcutaneous tissue were incised layer by layer until the ovaries were exposed in the abdominal cavity. In the Sham group, only the adipose tissue around the ovary was removed, while in the OVX-ed group, both fallopian tubes were ligated, and both ovaries were removed. After the operation, the abdominal cavity and wounds on both sides were sutured. All rats received postoperative prophylactic injection of penicillin (50,000 IU/kg/day) by intramuscular injection to prevent postoperative infection for 7 days. After 12 weeks of normal feeding, bilateral femurs were taken out from 2 rats in the Sham and OVX-ed groups, respectively, to verify the successful establishment of the model. Subsequently, 2 sham rats were used for model validation, and two rats were sacrificed, leaving 8 rats (Sham, n = 8). The rats in the OVX-ed group were randomly divided into three groups: ovariectomized surgery (OVX, n = 8), ovariectomy + daytime melatonin injection (OVX + DMLT, n = 9), and ovariectomy + nighttime melatonin injection (OVX + NMLT, n = 9). The ovariectomized rats were treated with an intraperitoneal injection of MLT (30 mg/kg/d) for 12 weeks [30]. MLT was injected at 9:00 a.m. in the OVX + DMLT group and 10:00 p.m. in the OVX + NMLT group. After 12 weeks of MLT treatment, all rats were euthanized by an overdose of pentobarbital (exceeding 200 mg/kg) administered by intraperitoneal injection and rat femurs, femoral medullary cavity content, and rat abdominal arterial blood were collected.

### Micro-CT

All femurs were scanned using high-resolution micro-CT (Quantum GX, PerkinElmer Co., Ltd., Hopkinton, MA, USA) under the same parameters. We considered 100 tomograms within 2 mm below the growth plate as our volume of interest (VOI) to ensure experimental consistency. Various parameters in the VOI (bone volume per total volume (BV/TV), trabecular bone number (Tb.N), mean connective density (Conn.D), and trabecular bone separation (Tb.Sp) were estimated [29].

### Biomechanical measurements

All right femur samples were immediately subjected to biomechanical testing after being extracted. All samples were fixed with resin to a metal base, with only the upper edge of the metal base exposed to a length of 20 mm of the femoral condyle shaft for biomechanical testing. The Universal Testing Machine (Instron 4501; Instron, Canton, MA, U.S.A.) was used to measure the maximum load-bearing capacity of the bone tissue and the deflection at the breakpoint until the bone tissue was deformed and broken. The Instron 4501 software was used to operate the crosshead and measure the maximum load-bearing capacity of the bone tissue and the deflection at the breakpoint. The crosshead speed for all the tests was set at 0.5 mm/min [31]. Data were automatically collected by The Instron 4501 software until the bone tissue was broken.

### Histology

After the fixed tissue was decalcified by EDTA, the tissue was incised and embedded in a wax block. The prepared paraffin blocks were sliced into 5-um-thick sections using a microtome (Leica Microtome, Wetzlar, Germany) and stained according to the manufacturer’s instructions for hematoxylin–eosin (HE) and Masson staining.5-um-thick sections were dewaxing by dewaxing solution and absolute ethanol. Then, Antigen retrieval is performed on the tissue on the section at 98 °C by antigen retrieval solution. The sections were incubated with 3% hydrogen peroxide at room temperature and in the dark for 25 min, in order to block endogenous peroxidase interference with the experiment. The sections were incubated in the primary antibody (TRAP/CD40L (TRAP, Abcam, ab65854, 1:1000), oseopontin (OPN, Abcam, ab214050, 1:1000) and melatonin receptors-1β (MT-1β, Abcam, ab229554, 1:1000), negative controls with PBS) overnight. After incubation of secondary antibodies HRP (Goat Anti-Rabbit/mouse IgG H&L, Abcam, ab205718/ab6789, 1:200) for 50 min, DAB chromogenic is performed. After DAB chromogenic is complete, hematoxylin stains the cell nucleus. Finally, after dehydration, the sealing gel is used… IHC results are interpreted by professionals, and 40 × field of view are selected for observation. OPN protein and TRAP protein were observed in 40 × field of the view of the growth plate and the surface of the growth plate. MT-1β was observed in the 40 × field of the growth plate and the surface of the growth plate. All IHC interpretations are based on the following standards, and the cells without expression were counted 0 point, low-expressing cells were counted 1 point, and high-expressing cells were counted 2 points. All data were statistically calculated after calculating the total score.

### Bone formation and resorption biomarker

The upper serum of reserved blood sample was used for the experiment. Blood retained at sacrifice was detected for N-terminal propeptide of procollagen type I (P1NP) and collagen type I cross-linked C-terminal peptide (CTX-I). The serum levels of P1NP (Immunodiagnostic Systems EURL, Paris, France), and CTX- I (Beijing Bright Biotechnology Co., Ltd, Beijing, China) were measured using EIA.

### Western blot

The contents of the medullary cavity pre-stored at − 80 °C were thawed, and the femoral canal content was lysed in a pre-made protein lysis buffer (RIPA: PMSF, 100:1) on ice. After 5 min, the cells were scraped using a cell scraper. The supernatant was collected by centrifugation at 14,000 rpm, 4 °C for 30 min to measure the protein content by the BCA method. The prepared samples were diluted and mixed with loading buffer, 1/4th of the volume of protein lysate. An equivalent of 20 ug/lane was separated by SDS-PAGE (sodium dodecyl sulfate–polyacrylamide gel electrophoresis) and transferred to a nitrocellulose membrane. Subsequently, the membranes were blocked with 5% bovine serum albumin (BSA) and probed with primary antibodies: runt-associated transcription factor 2 (RUNX2, Abcam, ab82336, 1:1000), Receptor Activator of Nuclear Factor Kappa B Ligand (RANKL, Abcam, ab239607,1:1000), osteoprotegerin (OPG, Abcam, ab73400,1:1000), and osteocalcin (OC, Abcam, ab93876, 1:1000) at 4 °C for 14 h, followed by incubation with the corresponding species of secondary antibody (1:5000, horseradish peroxidase anti-mouse and horseradish peroxidase anti-rabbit/anti-mouse). Finally, the protein expression was quantified by scanning autoradiography and densitometry using Image J (NIH).

### Cell culture

MC3T3-E1 cells are cultured in α minimum essential medium (α-MEM, Gibco), including 10% fetal bovine serum and 1% penicillin–streptomycin at 37 °C with 5% CO2. Hydrogen peroxide (H2O2, Beyotime, Shanghai) was purchased from Beyotime. The cells were subsequently classified into four experimental groups: (1) Control: the cells were only treated with α-MEM for 2 h; (2) H_2_O_2_: the cells were treated with α-MEM and H_2_O_2_ (400 μM) for 2 h; (3) H_2_O_2_ + LMLT: the cells were treated with α-MEM, low-dose MLT (100 μM) and H_2_O_2_ (400 μM) for 2 h; (4) H_2_O_2_ + HMLT: the cells were treated with α-MEM, high-dose MLT(400 μM) and H_2_O_2_ (400 μM) for 2 h.

### Cell viability assay

Cell survival rate was measured with the Cell Counting Kit 8 (CCK-8) colorimetric assay (Beyotime, Shanghai. China). Briefly, cells (5 × 10^3^) were cultivated in a 96-well plate. 10 μl CCK-8 assay solution was supplemented to 100 μl medium in every well, and incubated the cells in the dark for one hours at 37 °C before the cells were handled with different treatment(Previous plans). Then, the optical density of every well was got on a microplate reader (SpectraMax M5; Molecular Devices, CA, USA). Cell survival rate = The measured OD value/OD_control_*100%.

### Measurement of reactive oxygen species

The cells were cultivated into 6-well plates with a density of 1 × 105 cells/well for the whole day in three replicate wells. Then, the medium was replaced with α-MEM with or without H2O2 (400 μM) or melatonin (100 μM/400 μM) for 2 h. Levels of reactive oxygen species (ROS) were surveyed employing the fluorescent probe 2′,7′-dichlorofluorescein diacetate (DCFH-DA) (Beyotime, Shanghai, China). DCFH-DA, diluted to a density of 10 μM, was added to the cells, which then were hatched for 20 min at a temperature of 37 °C in the dark. The cells were measured by flow cytometry after being washed twice with Serum-free α-MEM.

### Cell apoptosis assay

5 × 10^4^cells/well MC3T3-E1 osteoblasts were seeded in a 6-well plate. Apoptosis was examined by Annexin V-fluorescein isothiocyanate staining. Briefly, centrifuge and collection MC3T3-E1 osteoblasts, then MC3T3-E1 osteoblasts were washed by PBS for three times. Then, 5 μL of Annexin-V-FITC and 5 μL of pyridine iodide were added and incubated for 30 min. Finally, the cells were measured by flow cytometry after being washed three times with PBS.

#### Statistical analysis

The statistical data are presented as mean ± standard deviation (SD). All data were checked for normality of variables using the Kolmogorov–Smirnov test and all data were distributed for normality. SPSS 22.0 software was used for statistical analysis; all data in this study were calculated using ANOVA test for mean and variance, and LSD post-hoc test was used to compare groups. *p* < 0.05 was regarded as a significant difference between groups. *p* < 0.05 indicated a significant difference [#represents statistical significance (*p* < 0.05) compared to the Sham group. *represents statistical significance (*p* < 0.05) compared to the OVX group. &represents statistical significance (*p* < 0.05) compared to the OVX + DMLT group].

## Results

### Animal experiments

Animal deaths did not occur during adaptive feeding. All animals were in a stable state during the operation and anesthesia, and the surgical results were satisfactory. Also, the rats were stably awake after the operation. Two rats died within 7 days after surgery. After dissecting the rat bodies, it was found that both rats had abdominal infections after surgery. None of the remaining animals died until sacrificed.

### Model identification

**Fig. 1:**
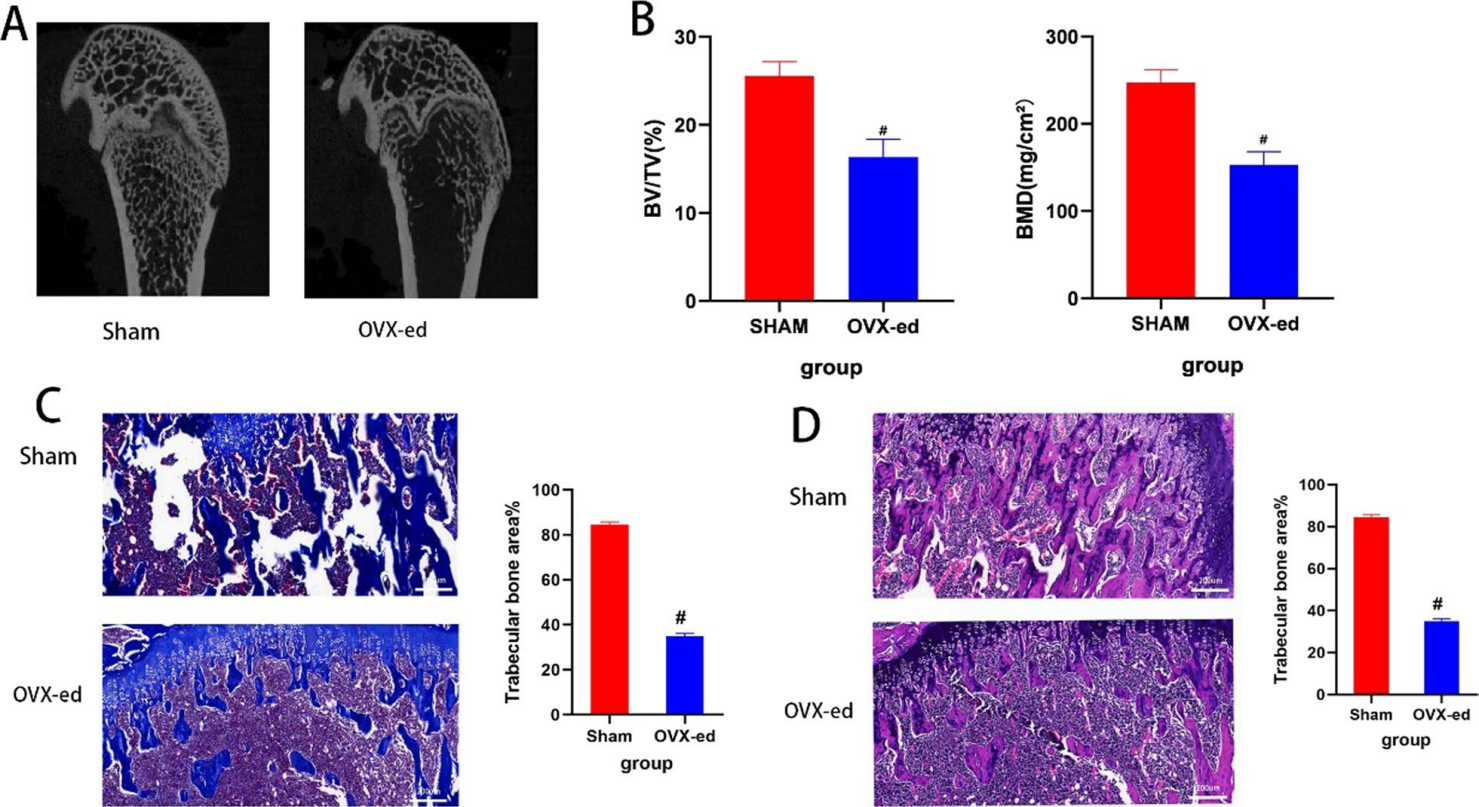
By analyzing the imaging and histological results, the bone mass of pre-OVX was significantly reduced. It was preliminarily judged that the menopausal bone loss model was successfully established. **A** Tomographic images of the rats in the Sham and pre-OVX groups. **B** Imaging parameters, BV/TV and BMD. **C** 10.0x: Masson staining results of the Sham and pre-OVX groups. **D** 10.0x: HE staining results of the Sham and the pre-OVX groups. #represents statistical significance (*p* < 0.05) compared to the Sham group

At 3 months after bilateral ovariectomy, a significant loss was observed in bone mass on the CT tomographic images in the OVX-ed group (Fig. 1A). As shown in Fig. 1B, bone mineral density (BMD) and BV/TV were significantly less than that of the Sham group (*p* < 0.05). Histologically, the results of HE and Masson staining showed that the density of connective tissue was significantly reduced in the OVX-ed (Fig. 1C, D). In conclusion, the model of this study was constructed successfully.

### Micro-CT

**Fig. 2:**
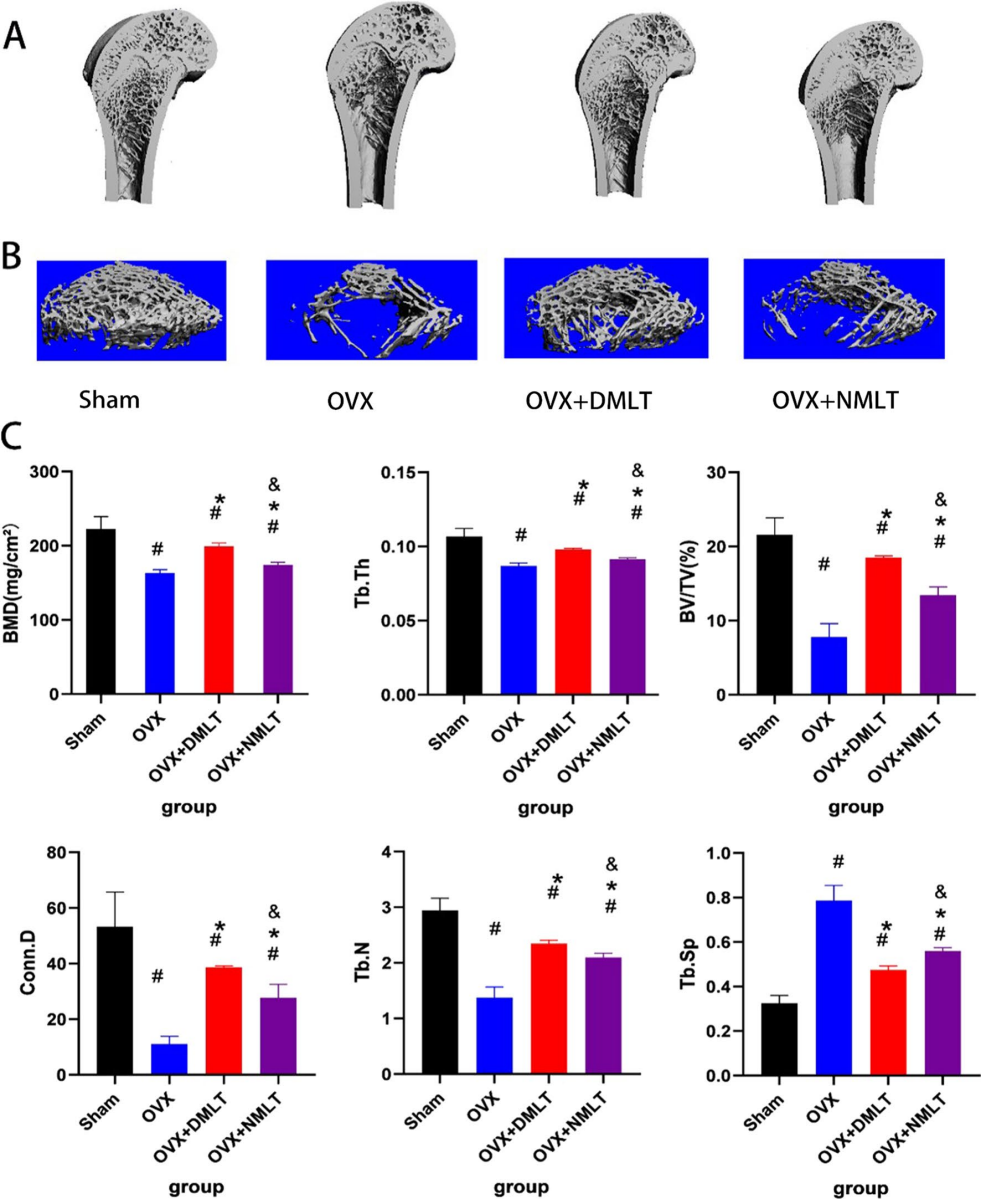
**A** 3D reconstruction results of the whole. **B** 3D reconstruction results of VOI of micro-CT. **C** Value of each imaging parameter and its comparative analysis. #represents statistical significance (*p* < 0.05) compared to the Sham group. *represents statistical significance (*p* < 0.05) compared to the OVX group. &represents statistical significance (*p* < 0.05) compared to the OVX + DMLT group

After 12 weeks of drug treatment, three-dimensional micro-CT reconstruction images showed that bone mass of distal femur of the two treated groups was significantly more than that of the OVX group (Fig. 2). The bone mass of OVX + DMLT group was significantly more than that of OVX + NMLT group. After measuring and analyzing BMD, Tb.Th, BV/TV, Tb.N, Conn.D, and Tb.Sp, the BMD, Tb.Th, BV/TV, Tb.N, and Conn.D of the OVX + DMLT and OVX + DMLT groups were significantly higher than those of the OVX group (*p* < 0.05). Among these, the BMD, Tb.Th, BV/TV, Tb.N, and Conn.D were significantly higher in the OVX + DMLT group than in the OVX + NMLT group (*p* < 0.05). The Tb.Sp of the OVX group was significantly higher than that of the other two treatment groups (*p* < 0.05). In the mutual comparison, the Tb.Sp of the OVX + DMLT group was significantly lower than that of the OVX + NMLT group (*p* < 0.05).

### Biomechanical testing

**Fig. 3:**
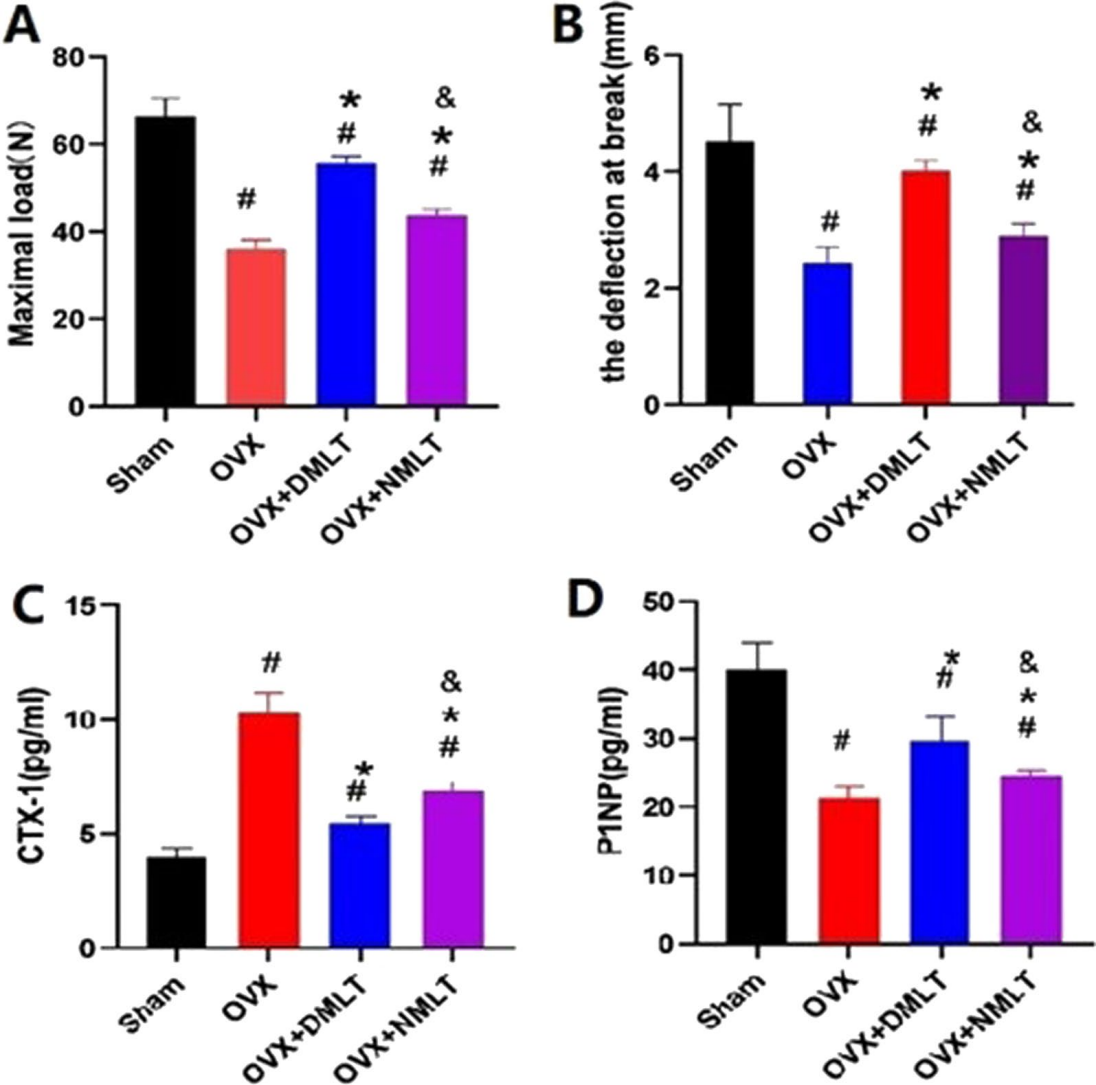
**A** Maximum weight-bearing capacity of each group. **B** Deflection at the break in each group. **C** Level of CTX-1 in the serum of each group and its comparative analysis. **D** Level of P1NP in the serum of each group and its comparative analysis. #represents statistical significance (*p* < 0.05) compared to the Sham group. *represents statistical significance (*p* < 0.05) compared to the OVX group. &represents statistical significance (*p* < 0.05) compared to the OVX + DMLT group

After 12 weeks of drug treatment, in the biomechanical test, the maximum weight-bearing capacity and deflection at break of the Sham group were the maximum values in each group (Fig. 3). The deflection at break and maximum load-bearing capacity of the two treatment groups were greater than those of the OVX group, and had statistical significance. The OVX + DMLT group had higher mechanical parameters than OVX + NMLT group, and it was statistically significant (*p* < 0.05).

### Bone formation and resorption biomarker analysis

Serum CTX-1 was significantly higher in the OVX group than in the Sham group (Fig. 3). Also, it was significantly higher than that in the OVX + DMLT and OVX + NMLT groups (*p* < 0.05) (Fig. 3). Strikingly, OVX + DMLT group showed a lower level than OVX + NMLT (*p* < 0.05). Serum P1NP was lowest in the OVX and statistically significant compared to the Sham group. However, it was lower in the OVX group than in the OVX + DMLT and OVX + NMLT groups (*p* < 0.05), while that in the OVX + DMLT group was higher than the OVX + NMLT group (*p* < 0.05).

### Histological evaluation

**Fig. 4:**
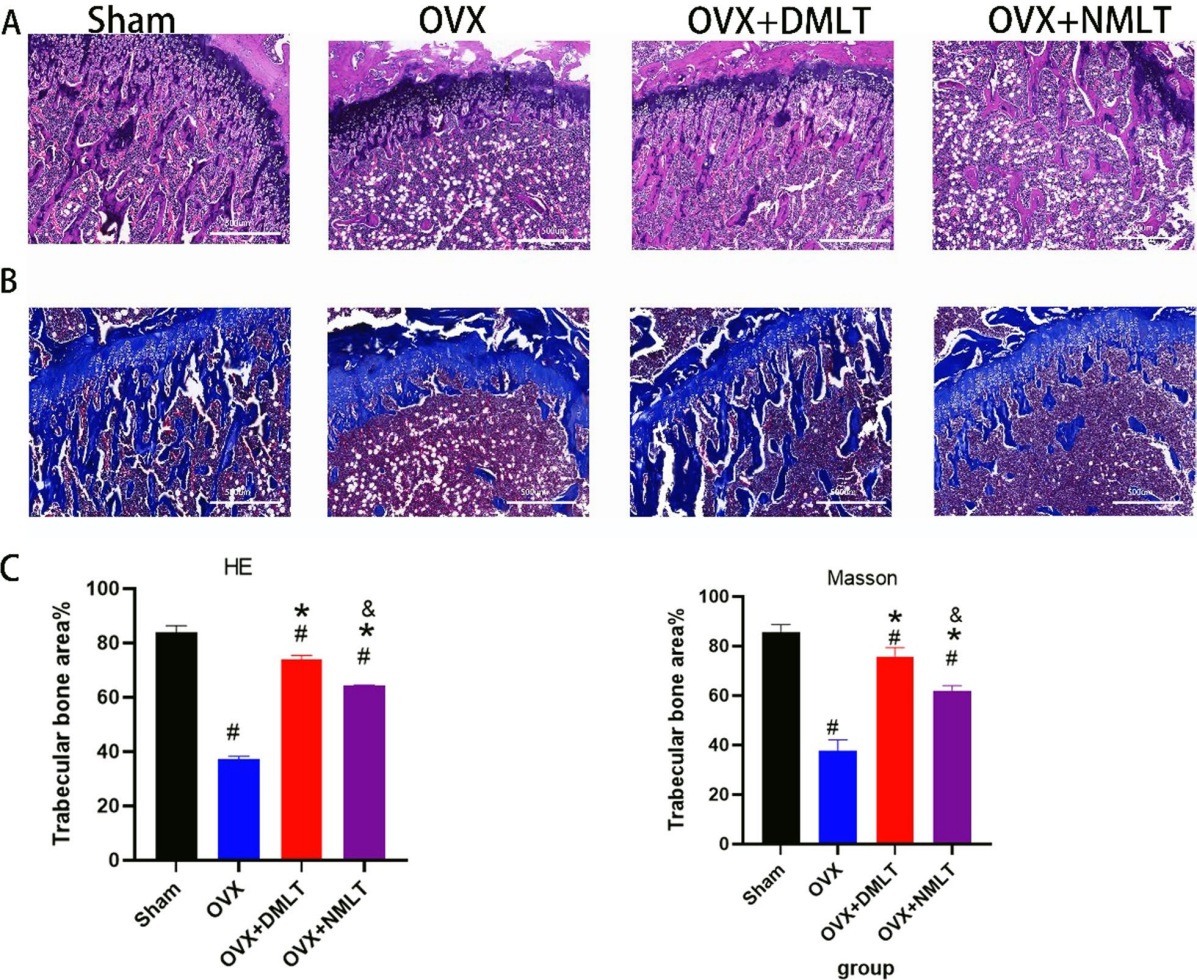
**A** Representative picture of HE staining of the rat bone tissue in each group, wherein the long red area stained with eosin indicates the trabecular bone. **B** Representative images of Masson staining of the rat bone tissue in each group; the blue area is the trabecular bone. **C** Histogram of trabecular bone as a percentage of total area #represents statistical significance (*p* < 0.05) compared to the Sham group. *represents statistical significance (*p* < 0.05) compared to the OVX group. &represents statistical significance (*p* < 0.05) compared to the OVX + DMLT group

**Fig. 5:**
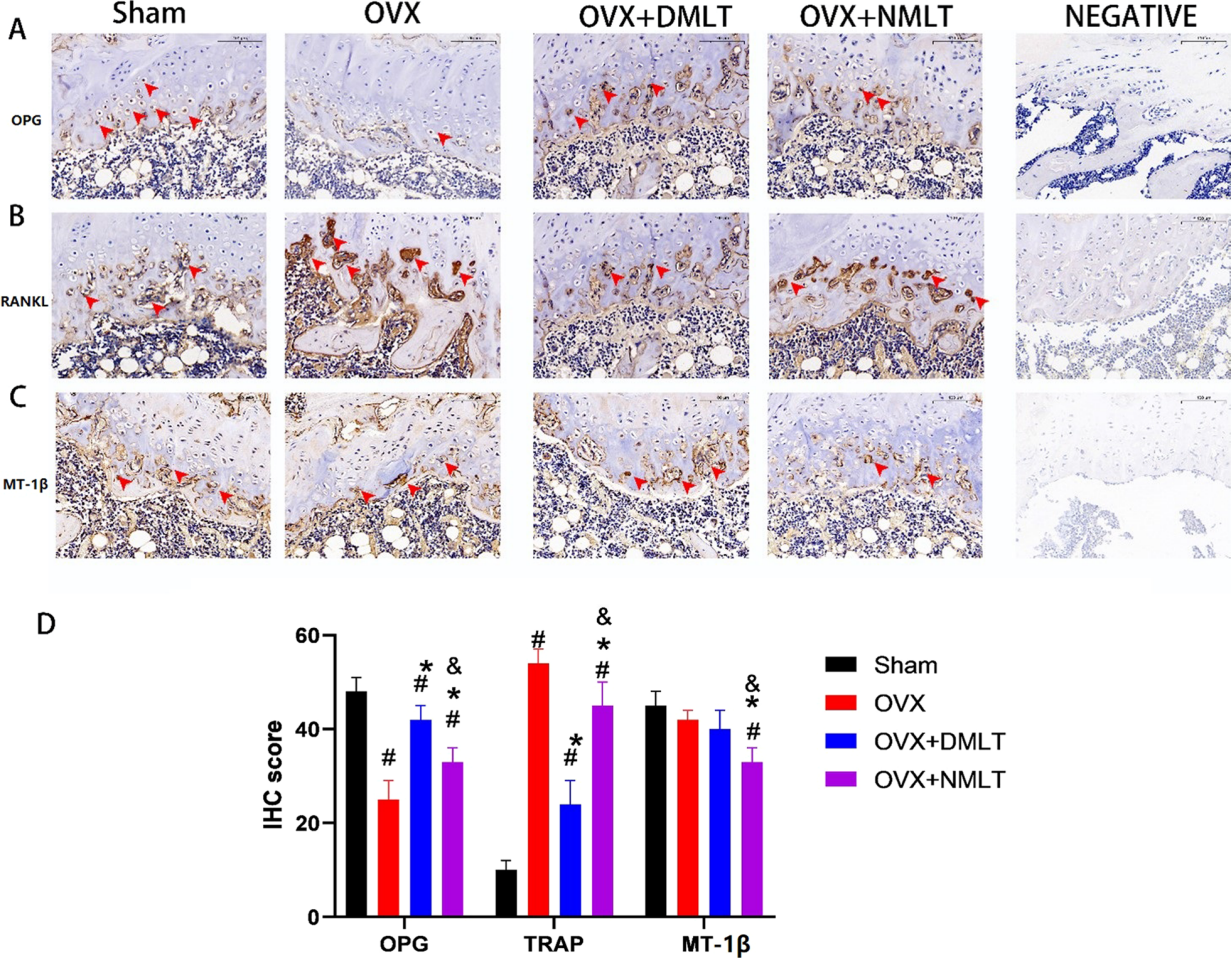
**A** 40x: Results of OPN immunohistochemical staining; arrows in the growth plate point to dark-brown-stained osteoblasts with high OPN protein expression. **B** 40X: Results of TRAP immunohistochemical staining; arrows in the growth plate point to dark-brown-stained osteoblasts with high TRAP protein expression. **C** 40X:Results of MT-1β immunohistochemical staining; arrows in the medullary cavity point to dark-brown-stained cell with high MT-1β expression. **D** IHC Interpretation Scores for each Proteins. #represents statistical significance (*p* < 0.05) compared to the Sham group. *represents statistical significance (*p* < 0.05) compared to the OVX group. &represents statistical significance (*p* < 0.05) compared to the OVX + DMLT group

In HE staining, it can be observed that the number of bone trabeculae in the Sham group is more and more dense, while the bone trabeculae in the OVX group are extremely sparse. After 12 weeks of melatonin treatment, denser trabecular bone was found in the OVX + DMLT and OVX + NMLT groups (Fig. 4). In Masson staining, similar to the results of HE staining, the number of bone trabeculae in the Sham group was the most dense, and the bone trabeculae in the OVX group was the most sparse. In the comparison of the OVX + DMLT group and the OVX + NMLT group, OVX + DMLT was more dense. In immunohistochemical staining, the expression of OPN was significantly increased in OVX + DMLT compared with OVX and OVX + NMLT. And the expression level of TRAP was significantly decreased, and it was statistically significant. No significant difference was found in the expression level of MT-1β among Sham, OVX and OVX + DMLT, but OVX + NMLT was significantly lower than the three groups with statistical significance (*p* < 0.05) (Fig. 5).

### Western blot

**Fig. 6:**
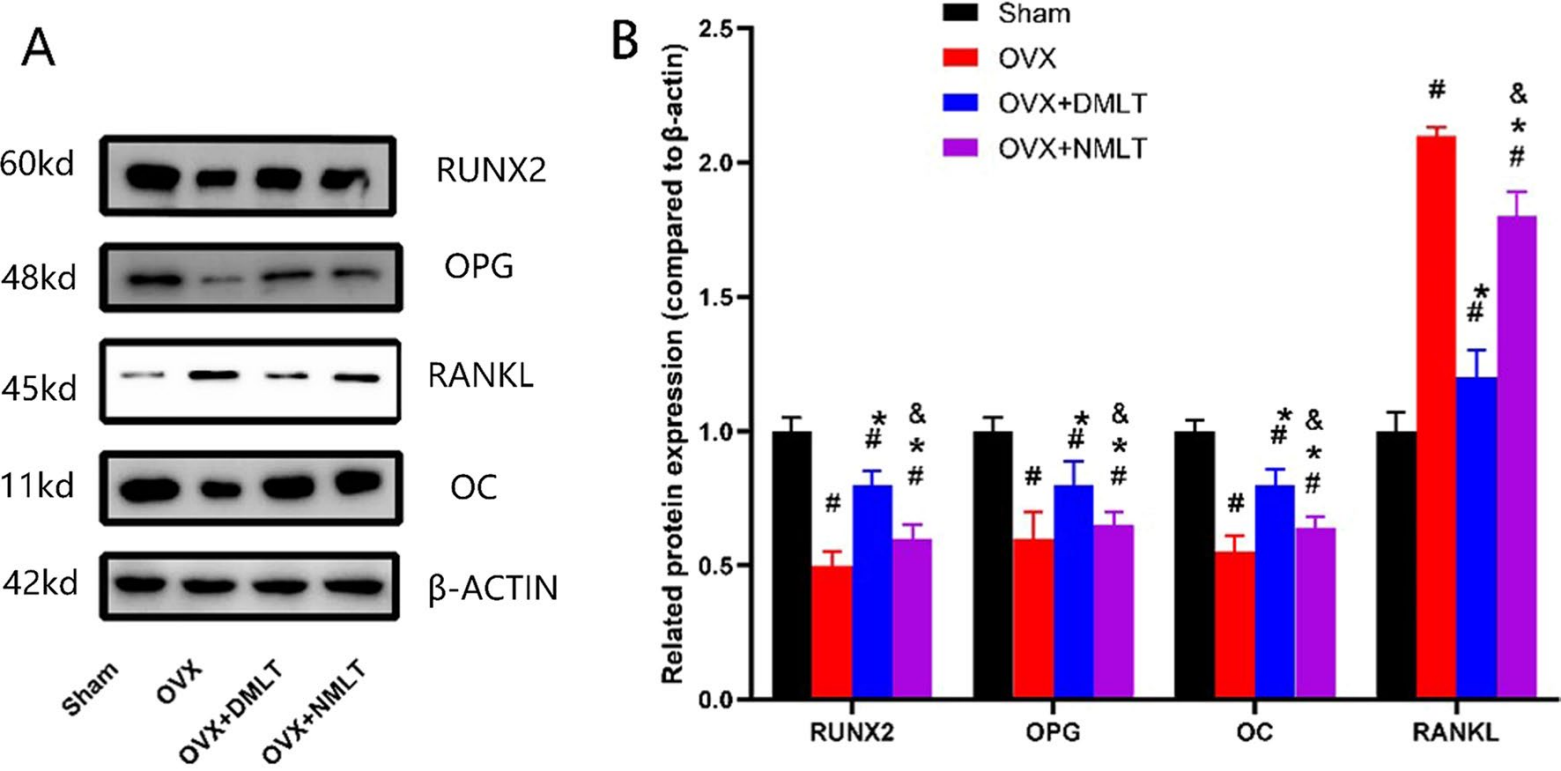
**A** Protein bands. **B** Statistical results of the expression levels of RUNX2, OPG, RANKL, and OC proteins compared to β-actin. #represents statistical significance (*p* < 0.05) compared to the Sham group. *represents statistical significance (*p* < 0.05) compared to the OVX group. &represents statistical significance (*p* < 0.05) compared to the OVX + DMLT group

The expression levels of osteogenesis-related proteins (OC, RUNX2, and OPG) in the OVX + DMLT group were significantly increased compared to OVX and OVX + NMLT groups (*p* < 0.05), while that of osteoclast-associated protein (RANKL) was significantly decreased (*p* < 0.05). The expression levels of OC, RUNX2, and OPG of OVX were the lowest, and that of RANKL was the highest in each group (Fig. 6).

### Cell viability assay

**Fig. 7:**
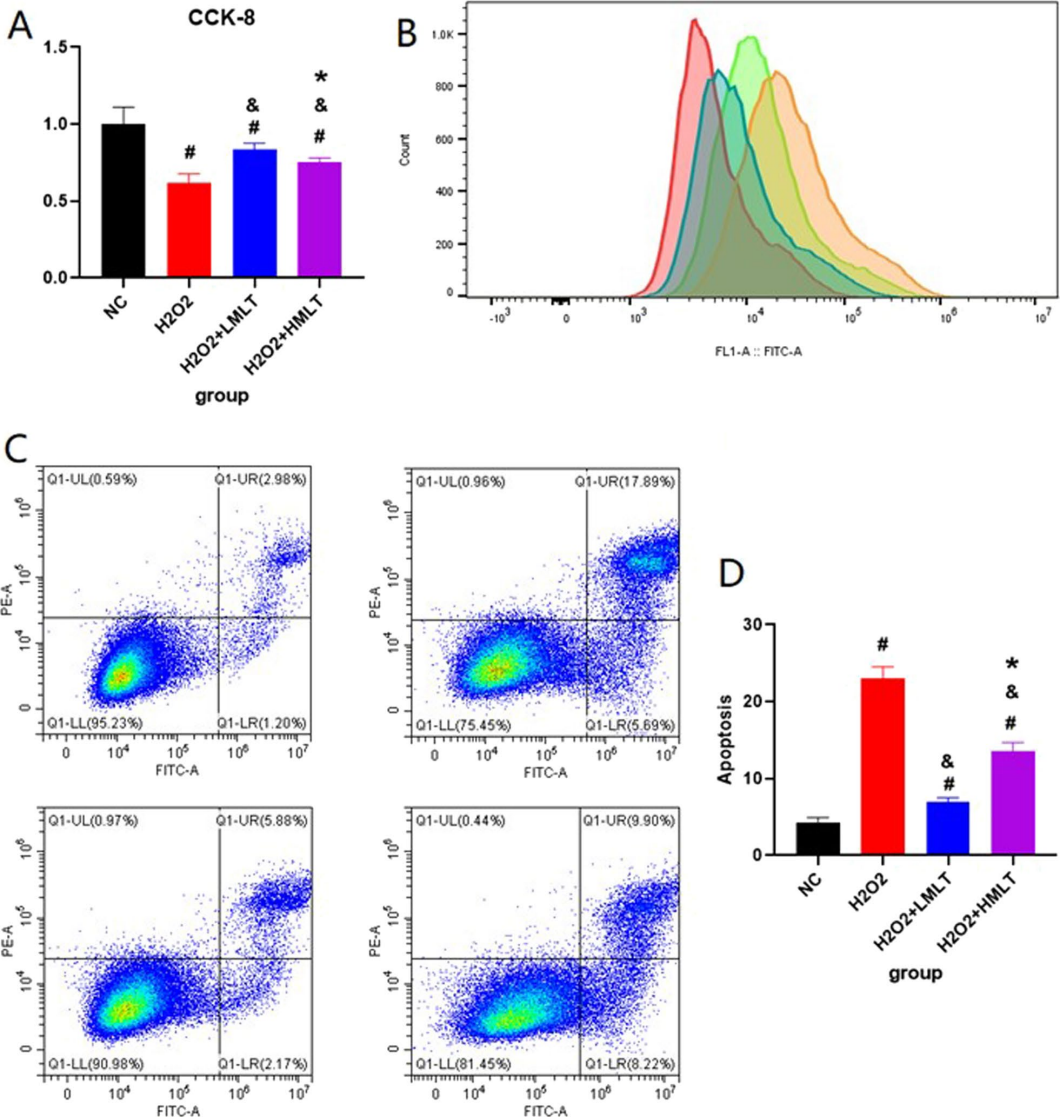
**A** CCK-8. **B** the measures of ROS. **C** Cell Aptosis. **D** The statistical results ot the Cell Aptosis. #represents statistical significance (*p* < 0.05) compared to the Sham group. *represents statistical significance (*p* < 0.05) compared to the OVX group. &represents statistical significance (*p* < 0.05) compared to the OVX + DMLT group

Cell viability results showed that both low-concentration and high-concentration MLT could effectively enhance the cell viability of H_2_O_2_-treated cells (*p* < 0.05). Compared with high concentrations of MLT, low-concentration MLT is more effective in increasing the cell viability of H_2_O_2_-treated cells (*p* < 0.05) (Fig. 7).

### Measurement of reactive oxygen species

ROS results show that both high-concentration MLT and low-concentration MLT can inhibit the accumulation of ROS (*p* < 0.05), and low-concentration MLT is more effective than high-concentration MLT (*p* < 0.05).

#### Cell apoptosis assay

The results of the cell apoptosis assay show both high-concentration MLT and low-concentration MLT can inhibit Cell apoptosis (*p* < 0.05), and low-concentration MLT is more effective than high-concentration MLT (*p* < 0.05) (Fig. 7).

## Discussion

Although the efficacy of MLT in the treatment of osteoporosis is definite, and its secretion as an endocrine hormone has a strong circadian rhythm and various secretion methods [32–35]. After exogenous MLT is injected into the body, whether the secretion of endogenous MLT is affected by the exogenous levels, effectuating varied anti-osteoporosis curative effects is yet to be elucidated. Therefore, the present study aimed to investigate the effect of MLT administration time on bone microstructural properties and BMD in OVX rats. In order to determine the drug effect of MLT in vivo, we employed animal experiments used in this study, which reflected the drug behavior intuitively and clearly. As demonstrated previously, MLT injections at various time points had different effects on the rats’bone mass. Interestingly, the daytime injection of MLT promotes trabecular bone formation and improves bone microarchitecture.

Menopausal osteoporosis is a disease of bone mass loss, bone microarchitecture destruction, and decreased bone strength caused by estrogen deficiency [36–38]. Low levels of estrogen cannot counteract the inflammatory response in the body, thereby aggravating the aging and apoptosis of osteoblasts and promoting the proliferation and differentiation of osteoclasts. This turn accelerates the bone remodeling cycle, severely affecting the normal bone metabolic balance [39]. In the present study, female SD rats were used to establish an osteoporosis model after bilateral ovariectomy to simulate the physiological state of estrogen deficiency in postmenopausal women, resulting in bone loss. In previous studies, we used this model, which ensured a high degree of surgical completion and low mortality [40, 41]. In this study, after 12 weeks post-bilateral ovariectomy, the loss of bone mass and the decrease in trabecular bone density was detected by micro-CT, HE staining, and Masson staining, indicating a high success rate and reproducibility of our bone loss models.

In order to obtain realistic results and an intuitive reflection of drug behavior, researchers have proposed the benefit of using ovariectomized animal models to stimulate bone loss-related studies [42, 43]. Herein, the injection of MLT into ovariectomized rats simulated the clinical use of MLT in postmenopausal women. We observed that MLT injection was effective in preventing bone loss. Moreover, bone mass and microarchitecture were significantly improved in the rats after treatment. Notably, BMD, commonly used to measure the changes in bone mass in patients, also increased after treatment (*p* < 0.05). Clinically, CTX-1 and P1NP are commonly used to evaluate the level of bone resorption and formation in patients [44]. The low levels of CTX-1 indicated reduced bone resorption in OVX + DMLT, while high levels of P1NP indicated higher levels of bone formation in the OVX + DMLT group. The OPG/RANKL signal pathway is the final mediator in the regulation of osteoclastogenesis [45]. RUNX2 binds to the vitamin D3 response element to promote the expression of OC, which then stimulates the formation of extracellular matrix and subsequently osteogenesis [46]. Osteprotegerin (OPG) is a key protein affecting bone resorption. The injection of MLT during the day can effectively promote the expression of OPG, which in turn binds RANKL [47]. At this time, RANKL cannot effectively activate osteoclast differentiation, resulting in a decrease in bone resorption in the body. On the other hand, TRAP is the main marker of osteoclasts, and low expression of TRAP in OVX + DMLT means a low level of bone resorption activity [48]. OPN molecule is a multifunctional soluble extracellular matrix-associated glycoprotein and has a specific arginine-glycine-aspartate (RGD) sequence, thus, it can be recognized, bound, and expressed by the corresponding integrin on the cell surface, making it important for cell adhesion and migration [47]. High levels of OPN demonstrate the high bone formation levels of OVX + DMLT. In addition, the level of RANKL protein in the OVX + DMLT group was significantly lower than that in the OVX + NMLT group. The maximum deflection at break and maximum load capacity of OVX + DMLT are relatively higher than those of OVX and OVX + NMLT. Thus, by detecting and analyzing the changes of the above indicators, the daytime injection of MLT can more effectively alter bone metabolic status to promote bone formation and inhibit bone resorption, and improve bone microscopic parameters and bone strength.

Based on previous findings, we speculated that although rodents are nocturnal animals, the role of MLT is not related to sleep and is closely related to wakefulness [49]. At night, endogenous MLT secretion becomes active. Thus, when MLT is injected at this time, the exogenous hormone increases the concentration of MLT in the blood and other tissues, resulting in a high concentration of MLT that has negative feedback in inhibiting the secretion of the pineal gland. The concentration of MLT in tissues was not increased, thereby not elevating the beneficial effects of MLT on bone mass. When the MLT was injected in the morning, the level of the hormone was low. After exogenous MLT injection, the in vivo levels returned to high concentration. The injection of MLT at this time could not induce the negative feedback mechanism of MLT in vivo, thereby facilitating its pharmacological behavior in vivo. This phenomenon increases the duration of the pharmacological effects of MLT in vivo. Secondly, considering that the concentration of MLT in the nighttime serum of the nighttime injection group is extremely high, the MLT receptor is affected by the high concentration of MLT, and the affinity and sensitivity of the MLT receptor to MLT are reduced, thereby limiting the drug effect. This may also be one of the reasons for the decreased efficacy of nighttime MLT injection. Taken together, these may be the reasons why MLT injections during the day are more beneficial in improving bone microarchitecture.

According to previous reports, the secretion of melatonin was fluctuating, showing a wave shape. This may be an evidence that there is a negative feedback regulation of MLT secretion [50]. Regrettably, before the rats were sacrificed in this study, the blood samples for a short time after the injection of MLT were not preserved, and it was impossible to directly prove through experiments that the nighttime injection of MLT could negatively feedback the secretion of endogenous melatonin. This is the shortcoming of this study. At the same time, we failed to use agonists and inhibitors of MT-1β receptors to prove that MT-1β receptors suffered from the side effects of high concentrations of MLT, resulting in decreased sensitivity to MLT. The above are the deficiencies in this study, which are also the future research directions of the authors of this study. MT-1β is an important link of MLT in regulating bone metabolism. MLT exerts pharmacological behavior by binding to MT-1β [51]. In order to explore whether the injection of MLT at different times will affect the MLT receptor, we performed IHC experiments to clarify the activity of the MT-1β receptor. Among all the group, we only found that the expression of MT-1β was decreased in OVX + DMLT. This phenomenon seems to suggest that high concentration of MLT at night can inhibit the affinity and sensitivity of MT-1β receptor to MLT.

Some studies have suggested that the pharmacological behavior of melatonin on oxidative stress may be not dose-dependent [52]. In order to determine whether MLT is dose-dependent, it is necessary to perform cell experiments to verify it. Excitingly, the results suggest that cell viability after high concentrations of MLT treatment decreased compared to cells treated with low concentrations of MLT in models of oxidative stress induced by H_2_O_2_. Among them, the results of ROS and apoptosis level detection experiments showed that low concentrations of MLT were more helpful in reducing ROS and apoptosis. This is consistent with previous research. Perhaps, melatonin may not be dose-dependent in terms of oxidative stress. These phenomena appear to be in favor of demonstrating that higher concentrations of melatonin at night may attenuate the pharmacological behavior of melatonin.

To the best of our knowledge, this is the first study on the effects of systemic administration of MLT on bone mass and bone density in bone loss models at different time points. Finally, combined with the phenomena we observed, our conclusions are as follows: 1. MLT can effectively reduce bone loss. 2. Daytime MLT injections are more effective in preventing bone loss than nighttime MLT injections. The reason for this phenomenon may be related to the pharmacological properties of MLT and the interference of endogenous melatonin in this study. (1) Exogenous MLT may negatively inhibit the secretion of endogenous melatonin. (2) Too excessive concentrations of MLT may have adverse effects on MT-1β. (3) When the concentration of melatonin is too high, it may affect the biological function of melatonin in the body. There are still some certain limitations in this article. (1) MLT concentration in rats was not measured in this study. (2) The negative feedback regulation mechanism of MLT secretion has not been proved by inhibition of pineal gland function in rats. The exploration of negative feedback regulation and MLT secretion mechanism is what the participants of this study need to explore in the next study. The aim of this study was to uncover the differences in the pharmacological behavior of daytime MLT injection and nighttime MLT injections, and provides a theoretical basis for the clinical treatment of bone loss.

## Data Availability

All data generated or analyzed during this study are included in this published article.
